# Bimetallic Gold-Silver Nanoparticles Supported on Zeolitic Imidazolate Framework-8 as Highly Active Heterogenous Catalysts for Selective Oxidation of Benzyl Alcohol into Benzaldehyde

**DOI:** 10.3390/polym10101089

**Published:** 2018-10-01

**Authors:** Lili Liu, Xiaojing Zhou, Yongmei Yan, Jie Zhou, Wenping Zhang, Xishi Tai

**Affiliations:** School of Chemistry & Chemical Engineering and Environmental Engineering, Weifang University, Weifang 261061, China; zhouxiaojing105@wfu.edu.cn (X.Z.); yanyongmei@wfu.edu.cn (Y.Y.); zhoujie1226@foxmail.com (J.Z.); zhangwenping1234@foxmail.com (W.Z.)

**Keywords:** metal-organic frameworks, core-shell, bimetallic catalyst, aerobic oxidation, benzyl alcohol

## Abstract

The metal-organic zeolite imidazolate framework-8 (ZIF-8) supported gold-silver bimetallic catalysts with a core-shell structure (Au@Ag/ZIF-8 and Ag@Au/ZIF-8) and cluster structure (AuAg/ZIF-8) were successfully prepared by the deposition-redispersion method. Energy dispersive X-ray spectroscopy (EDS) elemental mapping images displayed that in the Au@Ag/ZIF-8 catalyst, Ag atoms were deposited on an exposed Au surface, and core-shell structured Au@Ag particles with highly dispersed Ag as the shell were formed. Additionally, the XPS investigation at gold 4f levels and silver 3d levels indicated that the Au and Ag particles of Au@Ag/ZIF-8, Ag@Au/ZIF-8, and AuAg/ZIF-8 were in a zero valence state. Among the resultant catalysts obtained in this study, Ag@Au/ZIF-8 catalysts showed the highest catalytic activity for the selective oxidation of benzyl alcohol, followed by AuAg/ZIF-8 and Au@Ag/ZIF-8. The turnover frequency (TOF) values were in the order of Ag@Au/ZIF-8 (28.2 h^−1^) > AuAg/ZIF-8 (25.0 h^−1^) > Au@Ag/ZIF-8 (20.0 h^−1^) at 130 °C within 1 h under 8 bar O_2_ when using THF as solvent. The catalysts of Au@Ag/ZIF-8 and Ag@Au/ZIF-8 with core–shell structures have higher benzaldehyde selectivities (53.0% and 53.3%) than the AuAg/ZIF-8 catalyst (35.2%) in the selective oxidation of benzyl alcohol into benzaldehyde. The effect of the solvent, reaction temperature, reaction time, and reaction pressure on benzyl alcohol conversion and benzaldehyde selectivity in benzyl alcohol selective oxidation over Au@Ag/ZIF-8, Ag@Au/ZIF-8, and AuAg/ZIF-8 were also investigated. All of the catalysts showed excellent performance at 130 °C under 8 bar O_2_ within 1 h when using THF as the solvent in the selective oxidation of benzyl alcohol to benzaldehyde. Moreover, the catalysts can be easily recycled and used repetitively at least four times.

## 1. Introduction

The selective catalytic oxidation of benzyl alcohols to benzaldehyde is one of the most fundamental transformations both in the laboratory and in the industrial synthetic chemistry [1,2]. Benzaldehyde is an important intermediate and a high-value product in the cosmetic, food, dyestuff, pharmaceutical, and agrochemical industries [2,3,4]. The catalytic performance of supported monometallic and supported bimetallic catalysts such as Au, Pd, Ag, Au–Cu, Au–Pd, and Cu–Ni for the selective oxidation of benzyl alcohol has been extensively studied [4,5,6,7,8]. Among them, Au-based bimetallic catalysts have showed superior catalytic activity and selectivity in the selective oxidation of alcohols to aldehydes [8,9,10]. Sun et al. [8] found that the iron doped graphene (Fe–Gr) supported gold-palladium catalyst is beneficial to the generation of aldehydes in the selective oxidation of alcohols. The benzyl alcohol conversion and selectivity of benzaldehyde were 82.6% and 89.2% in the selective oxidation of benzyl alcohol at 110 °C under 0.3 MPa of O_2_ for 4 h using n-butanol as the solvent. However, the selectivity of ester was only 10.8% over the catalyst of Au–Pd/Fe–Gr. Due to the high price and limited resources of Au metal, a variety of catalyst preparation strategies have been developed to maximize Au utilization [11,12]. For example, core-shell structured catalysts with Au shells are considered to be promising alternatives to improve Au metal dispersion. Recently, Sun et al. [13] prepared Au@Pd/TiO_2_ catalysts by the two-step photodeposition method. Au@Pd(0.049)/TiO_2_ had the highest turnover frequency (TOF, 21961 h^−1^) for the catalytic oxidation of benzyl alcohol. Henning et al. [14] prepared Au–Pd core-shell nanocrystals by using a seed-mediated approach. The TOF and benzaldehyde selectivity are greater than 3250 min^−1^ and 95% over the Au–Pd core-shell nanocrystals with a shell thickness of 2.2 nm for the oxidation of benzyl alcohol.

The metal oxides (TiO_2_, MgO, MnO_2_, and Al_2_O_3_) [6,13,15], mesoporous silica [16], SBA-15 [17], and metal–organic frameworks (MOFs) [1] have been widely used as the support for the selective oxidation of alcohols. Among them, metal–organic frameworks (MOFs) are a new class of inorganic–organic hybrid materials that are composed of inorganic metal nodes and organic linkers [18,19]. With remarkably high porosity, high surface areas, and almost infinite synthetic tenability, MOFs have several advantages in catalysis over non-porous and zeolitic materials [20,21,22,23]. The high surface areas and controllable pore sizes of MOFs would facilitate the MOF supports to entrap various nanoparticles (NPs) [24,25,26]. In addition, the crystalline porous structure of MOFs would effectively limit the migration and aggregation of active catalytic metal nanoparticles, consequently making the nanoparticles that involved NPs/MOFs catalysts become highly active and reusable [27,28].

**Scheme 1:**
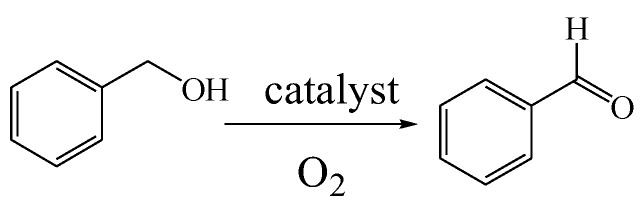
The oxidation of benzyl alcohol to benzaldehyde.

Zeolite imidazolate frameworks (ZIFs) are a subfamily of MOFs with extended three-dimensional structures from tetrahedral metal ions (e.g., Zn, Co) bridged by imidazolate linkers [29]. ZIF-8 (Zn(MeIM)_2_·2H_2_O, MeIM = 2-methylimidazole), a ZIF MOF, was always used as a support because of its high thermal stability (420 °C), large surface area (1400 m^2^/g), large porous diameter (around 11 Å), and convenient synthesis at room temperature [30,31,32]. Due to the presence of alkaline imidazole ligands, it has the potential to enhance the base-catalyzed reactions or be used as a basic support to favor metal dispersion [30,33]. In the present study, Au(core)-Ag(shell) (Au@Ag) and Ag(core)-Au(shell) (Ag@Au) nanoparticles were synthesized by the successive reduction method. For comparison, AuAg naoparticles were also obtained by simple physical mixing with Au nanoparticles and Ag nanoparticles. Then, the core-shell structured and cluster-structured catalysts Au@Ag/ZIF-8, Ag@Au/ZIF-8, and AuAg/ZIF-8 were prepared by the deposition-redispersion method. The catalytic activity of these catalysts was evaluated for the selective oxidation of benzyl alcohol into benzaldehyde using oxygen as an oxidant (Scheme 1). The results show that Ag@Au/ZIF-8 catalyst exhibited higher catalytic activity than Au@Ag/ZIF-8 and AuAg/ZIF-8 in the selective oxidation of benzyl alcohol into benzaldehyde, and core-shell structured catalysts Au@Ag/ZIF-8 and Ag@Au/ZIF-8 give higher benzaldehyde selectivity than cluster-structured catalyst AuAg/ZIF-8.

## 2. Materials and Methods

### 2.1. Materials and Chemicals

Zn(NO_3_)_2_·6H_2_O and 2-methylimidazole (H–MeIM) were purchased from Alfa-Aesar (Alfa-Aesar (China) Chemical Co., Ltd., Shanghai, China) and used as received. HAuCl_4_·4H_2_O and AgNO_3_ were obtained from Sigma-Aldrich (Sigma-Aldrich (Shanghai) Trading Co., Ltd., Shanghai, China) and used as supplied. Benzyl alcohol, hexadecyl trimethyl ammonium bromide (CTAB), L(+)-ascorbic acid, and sodium borohydride (NaBH_4_) were bought from Aladdin Chemistry Co., Ltd., Shanghai, China. Other chemicals such as tetrahydrofuran and ethanol were purchased from China National Medicines Co., Ltd., Beijing, China. All of the chemicals were analytical grade and used without further purification.

### 2.2. Catalyst Preparation

#### 2.2.1. Preparation of ZIF-8

ZIF-8 MOF was synthesized according to a previous report with slight modification [34]. Zn(NO_3_)_2_·6(H_2_O) (0.005 moL, 1.5 g) was firstly dissolved in 70 mL of methanol. Then, a 70-mL methanolic solution containing H-MeIM (0.04 moL, 3.3 g) was dropwise added to the mixture of Zn(NO_3_)_2_·6(H_2_O) under magnetic stirring at 500 rpm and continuously stirred for 24 h at room temperature. After the removal of mother liquor from the mixture by centrifugation (4000 r/min, 15 min), the resulting white crystals were washed thrice with methanol (15 mL × 3) to evacuate the guest molecules from the pores of ZIF-8. The crystals were finally dried at 80 °C for 12 h under vacuum in 0.1 MPa.

#### 2.2.2. Preparation of Catalyst

The procedure for Au@Ag/ZIF-8 was carried out as described below: (1) Synthesis of gold seed: CTAB (0.008 moL, 2.9 g) and HAuCl_4_·4H_2_O (10 mg) were mixed thoroughly in 78 mL of water until they were fully dissolved. Then, a 6-mL aqueous solution containing 0.06 mmoL NaBH_4_ was added dropwise to the above solution under vigorous agitation for about 30 min, and was then magnetically agitated at room temperature for 10 min. (2) Synthesis of Au nanoparticles: typically, 0.01 moL of CTAB was dissolved in 62 mL of H_2_O. Then, 12.5 mL at 10 mM of HAuCl_4_ solution was dropwise added to the solution of CTAB with constant vigorous stirring. After the mixture was stirred for a certain time, a 75-mL solution of 100 mM l(+)-ascorbic acid was added quickly into the mixture; then, a 0.6-mL gold seed solution was rapidly added into the mixture and continuously stirred for 10 min. (3) Synthesis of Au@Ag nanoparticles: 3.0 g of CTAB and 0.22 g l(+)- of ascorbic acid were dissolved in 62 mL of H_2_O at 50 °C. Then, the mixture was dropwise added into the sol of Au nanoparticles. Subsequently, 12.5 mL at 10 mM of AgNO_3_ was dropwise added to the above solution with constant vigorous stirring (500 rpm) at room temperature. After the mixture was stirred for 10 min, the Au@Ag nanoparticles were collected by centrifugation (RCF:13,000× *g*, 15 min) and washed thrice with ethanol (15 mL × 3). (4) Synthesis of Au@Ag/ZIF-8: the Au@Ag nanoparticles were redispersed in 3 mL of ethanol, and the mixture was sonicated for around 20 min until it became homogeneous. ZIF-8 (1.0 g) was added to the homogeneous mixture and continuously sonicated for another 1 h. Then, the as-synthesized sample was aged at room temperature for 12 h and dried under vacuum at 50 °C for 3 h to yield Au@Ag/ZIF-8. As a control, the catalyst Ag@Au/ZIF-8 was also prepared through similar procedures to those described above. The actual contents of Au and Ag in the catalysts have been determined by an inductively coupled plasma optical emission spectrometer (ICP–OES). The total gold and Ag contents in Au@Ag/ZIF-8 and Ag@Au/ZIF-8 were 1.2 and 1.1 wt %, and 0.8 and 1.0 wt %, respectively.

AuAg/ZIF-8 was also prepared by the deposition-redispersion method. First, 65 mL of Au nano gel was mixed with 65 mL of Ag nano gel; then, the AuAg nanoparticles were collected by centrifugation and washed thrice with ethanol (15 mL × 3). The AuAg nanoparticles were then redispersed in 3 mL of ethanol and sonicated for 20 min, followed by the addition of 1.0 g of ZIF-8 to the above homogeneous mixture. Then, the mixture was continuously sonicated for 1 h, stored at room temperature for 12 h, and dried under vacuum at 50 °C for 3 h to yield AuAg/ZIF-8. The gold and silver contents of AuAg/ZIF-8 were 0.9 and 1.1 wt %, respectively, as determined by ICP-OES.

### 2.3. Characterization

X-ray diffraction (XRD) analysis was performed on a Brüker D8 Advance diffractometer (Karlsruhe, Germany) with Cu Kα radiation (λ = 0.15406 nm). XRD patterns were obtained in the range from 5° to 70° with a step size of 0.02°. Nitrogen adsorption/desorption measurement was performed on a Quantachrome surface area instrument at 77 K (Boynton Beach, FL, USA). Prior to analysis, the samples were outgassed overnight at 150 °C under vaccum in 4 Pa. The morphologies and particle sizes of the as-prepared catalysts were observed by transmission electron microscopy (TEM) using a JEOL-2100F electron microscope (Jeol, Japan) with a high-angle annular dark-field (STEM-HAADF) detector. Energy dispersive X-ray spectroscopy (EDS) elemental mapping was recorded using an Oxford X-MaxN 80T IE250 instrument (Oxford, UK). Samples were prepared by ultrasonic dispersion in ethanol with a drop of the resultant suspension evaporated onto a holey carbon-supported grid. The Au and Ag contents in the catalysts were quantitated by an inductively coupled plasma optical emission spectrometer (ICP-OES) on a Perkin-Elmer Optima 7000 DV instrument (Waltham, MA, USA). Prior to analysis, the catalysts (20 mg) were first digested in aqua regia (HCl/HNO_3_) or nitric acid (HNO_3_), and further diluted with deionised water. X-ray photoelectron spectroscopy (XPS) data were obtained on an AXIS ULTRADLD (Shimadzu, Japan) with Al Kα radiation (*h*_υ_ = 1486.6 eV).

### 2.4. Catalyst Testing

The selective oxidation of benzyl alcohol to produce benzaldehyde was performed in a 10-mL stainless steel high-pressure reactor equipped with magnetic stirring and a temperature controller. In a typical experiment, benzyl alcohol (0.2 mmoL, 21.6 mg), tetrahydrofuran (THF, 1.5 mL), and catalysts (40 mg) were added into the 10-mL stainless steel autoclave. After the reactor was sealed, the pure O_2_ was pumped to replace the atmosphere five times. Subsequently, the reactor was kept in an oil bath at 120–140 °C for certain times under the pressure of 6–10 bar with stirring at 500 rpm. After the reaction, the reactor was thoroughly cooled down to room temperature to avoid the loss of the substrate, and the O_2_ were evacuated from the reactor via a cut-off valve before collecting the mixture. Then, the catalyst was separated by centrifugation (RCF: 13,000× *g*, 6 min). The reaction mixture was analyzed by using a gas chromatograph (GC-6890, Purkinje General instrument Co., Ltd., Beijing, China) equipped with a SE-54 capillary column and a flame ionization detector (FID, Purkinje General instrument Co., Ltd., Beijing, China). The products were identified by comparison with known authentic standards, and an external standard method was used for the qualitative analysis. Catalysts were recycled after the catalytic reactions. The catalyst was separated by centrifugation, washed with ethanol (2 × 3 mL), and dried under vacuum in 0.1 MPa at 40 °C for 3 h.

## 3. Results and Discussion

### 3.1. Catalyst Synthesis and Characterization

**Scheme 2:**
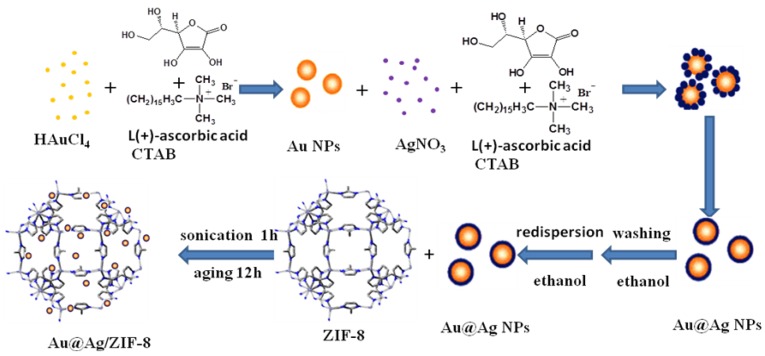
The strategy for the Au@Ag/zeolite imidazolate framework Zn(2-methylimidazole)_2_·2H_2_O (ZIF-8) catalyst by the deposition-redispersion method.

**Scheme 3:**
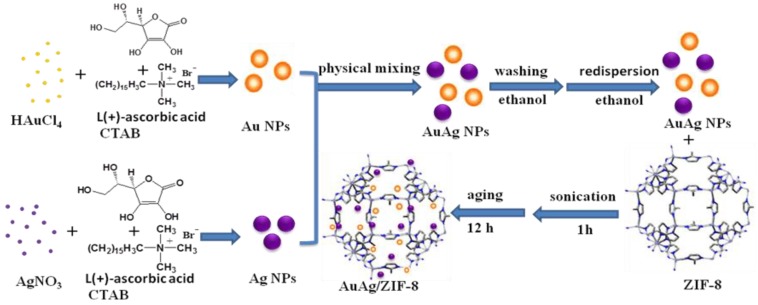
The strategy for the AuAg/ZIF-8 catalyst by the deposition-redispersion method.

Scheme 2 and Scheme 3 show the strategy for the Au@Ag/ZIF-8 and AuAg/ZIF-8 catalysts by the deposition-redispersion method. Two steps have been used to synthesize the catalysts of Au@Ag/ZIF-8, Ag@Au/ZIF-8, and AuAg/ZIF-8. Firstly, the core-shell structured Au@Ag and Ag@Au nanoparticles and cluster-structured AuAg nanoparticles were synthesized with the precursors of HAuCl_4_·4H_2_O and AgNO_3_ by the successive reduction method and physical mixing method, respectively. l(+)-ascorbic acid and CTAB were used as the reduction and stabilizing agents, respectively. The Au@Ag, Ag@Au, and AuAg nanoparticles were further washed using ethanol to remove the remaining l(+)-ascorbic acid and CTAB on the surface of the nanoparticles. These nanoparticles were then redispersed in the ethanol under ultrasonic. In addition, the nanoparticles were deposited on the ZIF-8 by the deposition-redispersion method. Traditionally, these reduction and stabilizing agents are removed by using high temperature [35]. However, the structures of the nanoparticles can seriously destroyed by conventional high temperatures [35]. The deposition–redispersion method could avoid the destroying of the nanoparticles’ structure.

**Figure 1:**
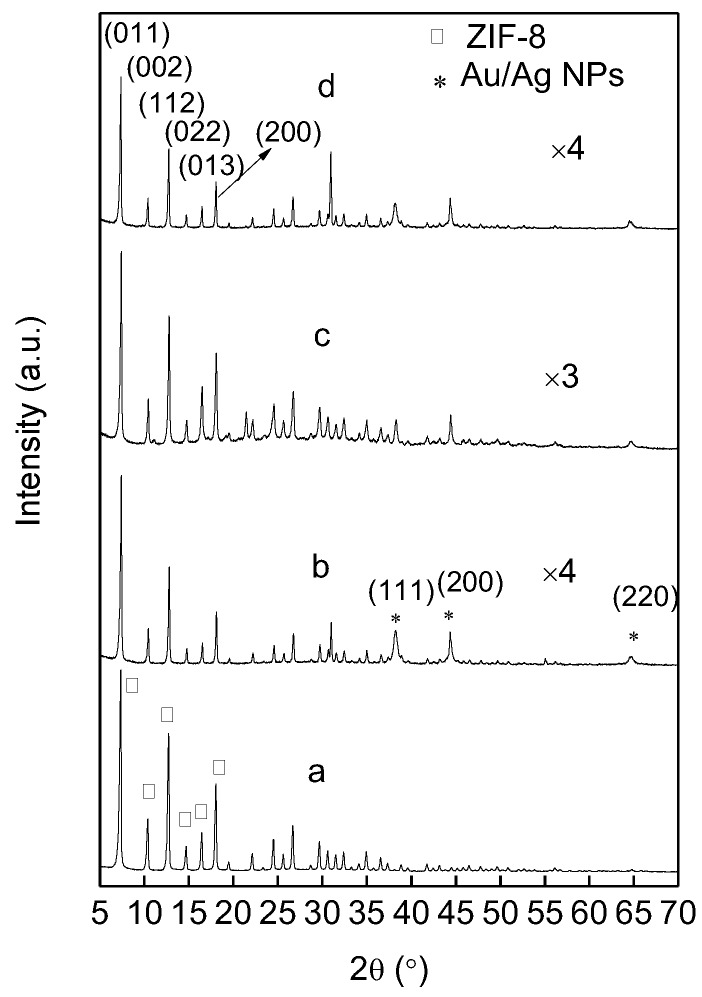
XRD patterns of ZIF-8 (**a**), Au@Ag/ZIF-8 (**b**), Ag@Au/ZIF-8 (**c**), and AuAg/ZIF-8(**d**).

Figure 1 shows the XRD patterns of ZIF-8, Au@Ag/ZIF-8, Ag@Au/ZIF-8, and AuAg/ZIF-8. The diffraction peaks of the as-prepared ZIF-8 particles were consistent with those reported in the literature [36,37], indicating that the obtained particles are indeed ZIF-8 crystals with sodalite (SOD) topology. Obviously, the diffraction peaks of Au@Ag/ZIF-8, Ag@Au/ZIF-8, and AuAg/ZIF-8 were all kept intact while their peak intensities became weaker, which indicated that the structures of ZIF-8 remained intact after gold-silver nanoparticles loading, but their crystallinity have been declined. The catalysts of Au@Ag/ZIF-8, Ag@Au/ZIF-8, and AuAg/ZIF-8 exhibited three characteristic diffraction peaks at angles 2θ = 38.3°, 44.3°, and 64.7° (JCPDS 01-1174), which are indexed to (111), (200), and (220) planes, respectively, for the face-centered cubic (FCC) lattice structure of metallic Au. As reported, the space lattice of the Au(111) and Ag(111) planes match each other (0.408 and 0.409 nm for Au and Ag); their diffraction patterns are overlapped at 38.3° (2θ) [38,39]. This shows that the Au@Ag, Ag@Au, and AuAg particles are successfully attached on ZIF-8.

**Figure 2:**
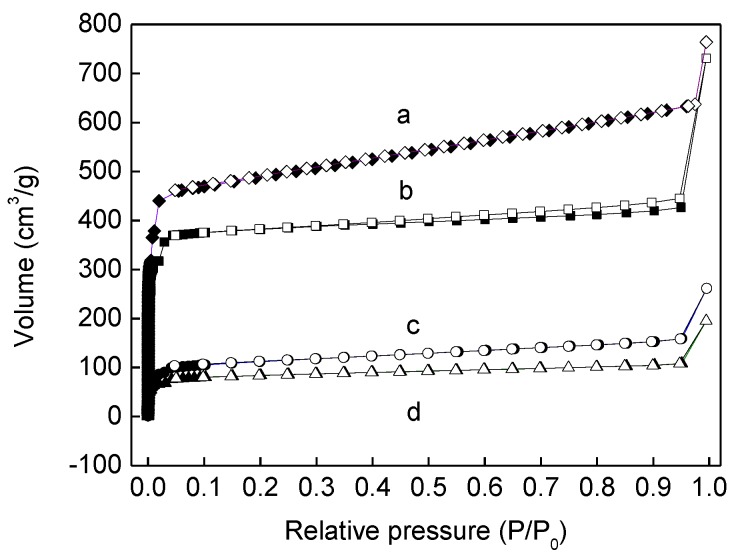
Nitrogen sorption isotherms of samples measured at 77 K: (**a**) ZIF-8; (**b**) AuAg/ZIF-8; (**c**) Au@Ag/ZIF-8; (**d**) Ag@Au/ZIF-8.

**Figure 3:**
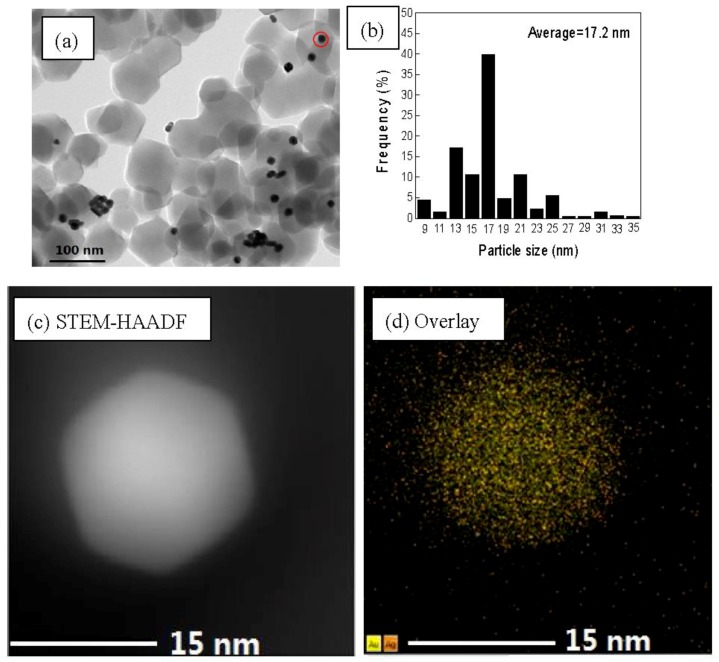

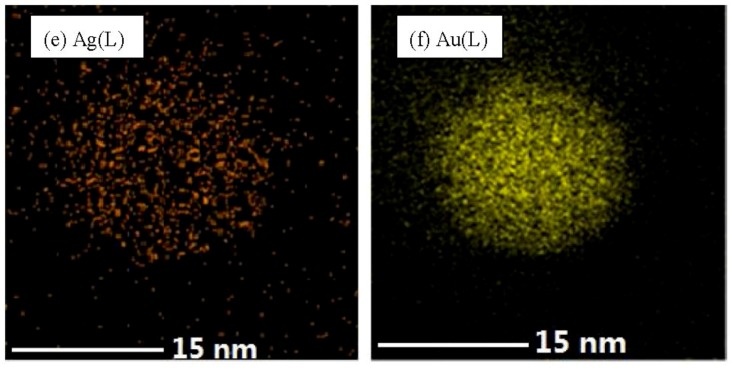
(**a**) TEM photograph (black spots represent the Au@Ag nanoparticles) and (**b**) Au@Ag size distribution of Au@Ag/ZIF-8. (**c**) STEM-high-angle annular dark-field (HAADF) image (selected one of Au@Ag nanoparticles in red circles) and (**d**–**f**) Energy dispersive X-ray spectroscopy (EDS) elemental mapping images of Au@Ag nanoparticles (NPs).

**Figure 4:**
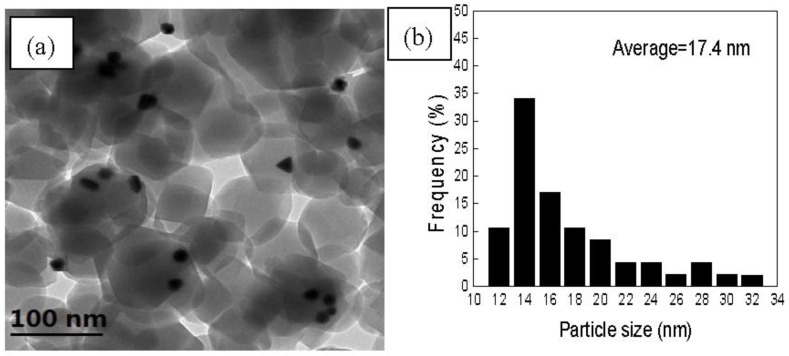
TEM photograph (**a**); black spots represent the Ag@Au nanoparticles) and size distribution of Ag@Au/ZIF-8 (**b**).

**Figure 5:**
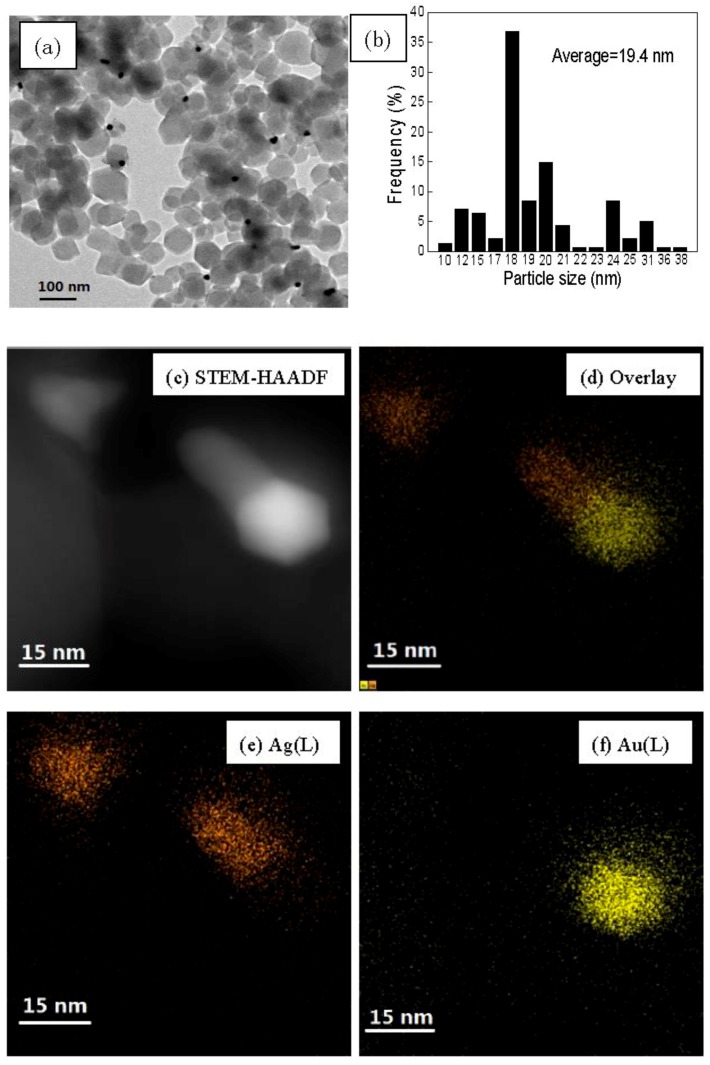
(**a**) TEM photograph (black spots represent the AuAg nanoparticles) and (**b**) AuAg size distribution of AuAg/ZIF-8. (**c**) STEM-HAADF image and (**d**–**f**) EDS elemental mapping images of AuAg NPs.

The nitrogen adsorption-desorption isotherms of the ZIF-8, AuAg/ZIF-8, Au@Ag/ZIF-8, and Ag@Au/ZIF-8 are displayed in Figure 2. All of the samples display type-І adsorption-desorption isotherms, which are characteristic of microporous materials [40]. The Brunner–Emmet–Teller (BET) surface areas of ZIF-8, AuAg/ZIF-8, Au@Ag/ZIF-8, and Ag@Au/ZIF-8 were 1763, 1530, 433, and 331 m^2^/g, respectively. The specific surface area for ZIF-8 significantly decreased upon further AuAg, Au@Ag, and Ag@Au particles loading. The appreciable decrease in the surface area suggests that the pores of the host framework ZIF-8 are occupied by AuAg, Au@Ag, and Ag@Au particles or/and blocked by AuAg, Au@Ag, and Ag@Au particles, which are located at the surface [41]. In addition, the BET surface area of AuAg/ZIF-8 is much higher than Au@Ag/ZIF-8 and Ag@Au/ZIF-8. This could be due to the nanopaticles of Au@Ag and Ag@Au being smaller than AuAg nanoparticles; Au@Ag and Ag@Au are more accessible to the cavities of ZIF-8 (Figure 3b, Figure 4b and Figure 5b).

Figure 3a,b shows a TEM photograph and size distribution of Au@Ag/ZIF-8, revealing that Au@Ag nanoparticles have an average particle size of 17.2 nm. Figure 3c shows STEM-HAADF images of Au@Ag nanoparticles. For the sake of the brightness being approximately proportional to the square of the atomic number (Z^2^) in a STEM-HAADF image, heavier Au atoms (atomic number *Z* = 79) give rise to a brighter image than lighter Ag atoms (*Z* = 49) [39]. As shown in Figure 3d–f, EDS elemental mapping images revealed that the Au core is uniformly covered by an Ag shell. This confirmed that the preparation method proposed by us could generate core-shell structure Au@Ag particles with highly dispersed Ag as the shell. Figure 4 shows a TEM photograph and the size distribution of Ag@Au/ZIF-8. As shown in Figure 4, the Ag@Au/ZIF-8 catalyst has an average particle size of 17.4 nm. Although the average particle size of the Ag@Au/ZIF-8 (17.4 nm) is nearly identical to that of Au@Ag/ZIF-8 (17.2 nm), the Ag@Au/ZIF-8 catalyst has a narrower particle size distribution (the majority of the particle sizes were between 12–20 nm) unlike the Au@Ag/ZIF-8 catalyst. Figure 5 gives a TEM photograph that indicates the size distribution of AuAg/ZIF-8, STEM-HAADF, and EDS elemental mapping images of AuAg nanoparticles. The average particle size (19.4 nm) of AuAg/ZIF-8 is slightly bigger than both Au@Ag/ZIF-8 (17.2 nm) and Ag@Au/ZIF-8 (17.4 nm). EDS elemental mapping patterns revealed that Au and Ag in the catalyst of AuAg/ZIF-8 existed as individual nanoparticles.

**Figure 6:**
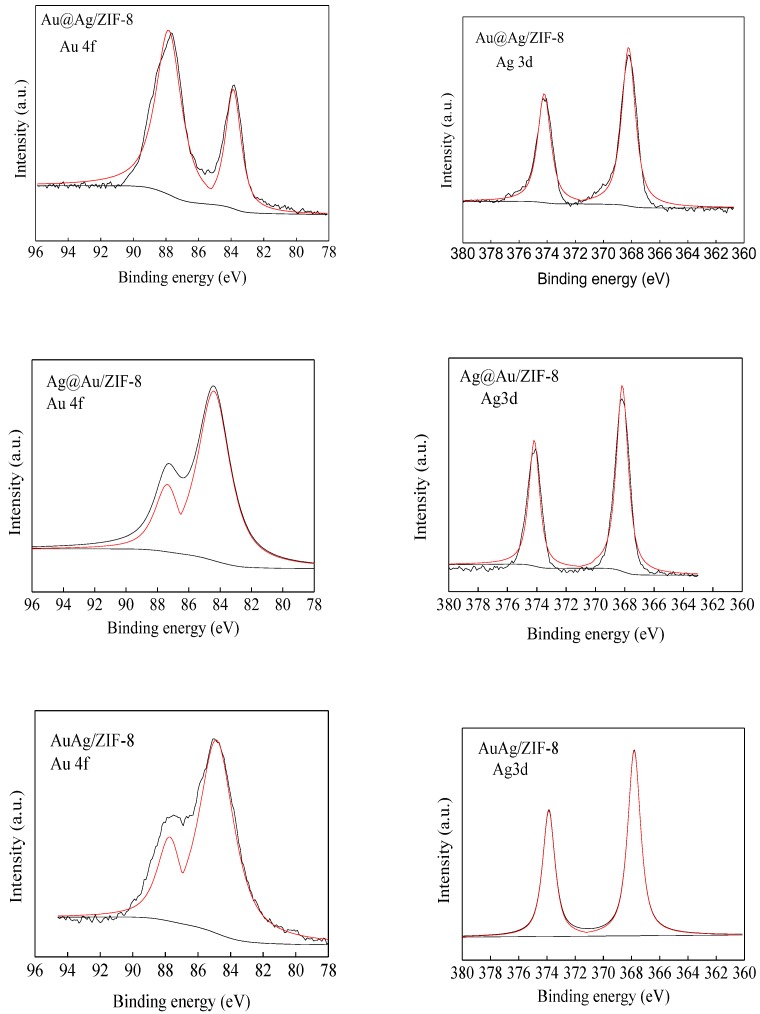
X-ray photoelectron spectroscopy (XPS) spectras of Au@Ag/ZIF-8, Ag@Au/ZIF-8, and AuAg/ZIF-8.

To clarify the valence state of Au and Ag in the catalysts of Au@Ag/ZIF-8, Ag@Au/ZIF-8, and AuAg/ZIF-8, XPS measurements were carried out. Figure 6 shows the Au 4f and Ag 3d XPS spectras of Au@Ag/ZIF-8, Ag@Au/ZIF-8, and AuAg/ZIF-8. Two peaks located at the range of 87.3–87.8 eV and 83.6–84.5 eV were observed in Au@Ag/ZIF-8, Ag@Au/ZIF-8, and AuAg/ZIF-8, with the peaks being attributed to typical of Au^0^ 4f_5/2_ and 4f_7/2_, respectively [42]. The XPS spectras of the present investigation did not show any peaks corresponding to the binding energies at 85 and 89 eV due to the cationic form of Au in +3 oxidation states. For all of the samples, the spectras recorded in the Ag 3d region are dominated by the two peaks centered at ca. 374.2 and 368.2 eV, which are generally ascribed to Ag^0^ 3d_3/2_ and 3d_5/2_, respectively [43,44]. The XPS investigation at gold 4f levels and silver 3d levels indicated that Au and Ag particles on the surface of the ZIF-8 support are in a zero valence state. The samples of Au@Ag/ZIF-8, Ag@Au/ZIF-8, and AuAg/ZIF-8 contain metallic Au^0^ and Ag^0^ species.

### 3.2. The Selective Catalytic Oxidation of Benzyl Alcohol

**Figure 7:**
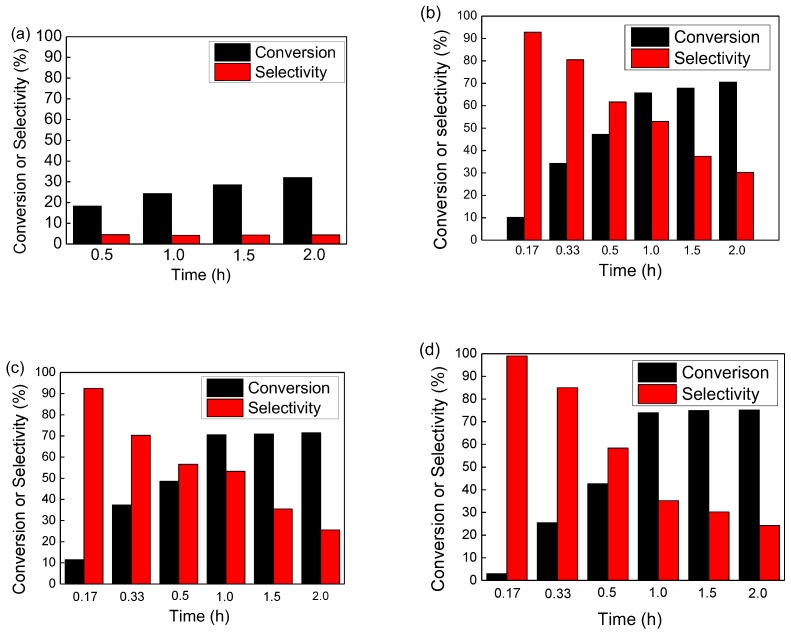
The conversion of benzyl alcohol and selectivity of benzaldehyde in the oxidation of benzyl alcohol at 130 °C under 8 bar in THF over ZIF-8 (**a**), Au@Ag/ZIF-8 (**b**), Ag@Au/ZIF-8 (**c**), and AuAg/ZIF-8 (**d**).

The catalytic performances of Au@Ag/ZIF-8, Ag@Au/ZIF-8, and AuAg/ZIF-8 were evaluated for the selective oxidation of benzyl alcohol to benzaldehyde with oxygen as the oxidant, which is a reaction that is often used as a model reaction to check the catalytic performance of metal nanoparticles [45,46]. Initially, the effect of reaction time was studied to optimize the reaction condition. The conversion of benzyl alcohol and selectivity of benzaldehyde in the oxidation of benzyl alcohol at 130 °C under 8 bar O_2_ in THF over ZIF-8, Au@Ag/ZIF-8, Ag@Au/ZIF-8, and AuAg/ZIF-8 are shown in Figure 7. A blank experiment shows that ZIF-8 support without gold-silver particles loading exhibits a low benzyl alcohol conversion (24.3%) and a negligible benzaldehyde selectivity (<5%) at 130 °C under 8 bar O_2_ using THF as the solvent within 1 h (Figure 7a). It can be seen that the reaction time has a remarkable effect on the benzyl alcohol conversion and benzaldehyde selectivity for the bimetallic gold-silver supported catalysts. As the reaction time increases, the conversion of benzyl alcohol increases over the catalysts of Au@Ag/ZIF-8, Ag@Au/ZIF-8, and AuAg/ZIF-8. However, the selectivity toward benzaldehyde decreased with the increasing reaction time. The benzyl alcohol conversions were 65.7%, 75.3%, and 74.0% at 130 °C under 8 bar O_2_ within 1 h over Au@Ag/ZIF-8, Ag@Au/ZIF-8, and AuAg/ZIF-8, respectively. The turnover frequency (TOF) values are calculated to be 20.0, 28.2, and 25.0 h^−1^ based on the total metal content of the catalyst for Au@Ag/ZIF-8, Ag@Au/ZIF-8, and AuAg/ZIF-8, respectively. The highest catalytic activity was obtained with the core-shell structured Ag@Au/ZIF-8 synthesized by the deposition-redispersion method, followed by AuAg/ZIF-8 and Au@Ag/ZIF-8. This can be attributed to the Ag@Au/ZIF-8 catalyst having a narrower particle size distribution, as shown in Figure 3 and Figure 4 [15,47]. Miedziak et al. [47] synthesized bimetallic Au–Pd supported catalysts 1%(Au–Pd)/TiO_2Im_, 5%(Au–Pd)/TiO_2DPCw_, and 1%(Au–Pd)/TiO_2SIm_ by the impregnation, deposition-precipitation, and sol-immobilization methods, respectively. The benzyl alcohol conversions and benzaldehyde selectivities were 16.8% and 70.6%, 19.2% and 80.6%, and 29.1% and 91.6% over 1%(Au–Pd)/TiO_2Im_, 5%(Au–Pd)/TiO_2DPCw_, and 1%(Au–Pd)/TiO_2SIm_ at 100°C under 10 bar O_2_ within 4 h for the oxidation of benzyl alcohol, respectively. The 1%(Au–Pd)/TiO_2SIm_ catalyst synthesized by the sol-immobilization method with a smaller particle size (around 4 nm) and a narrower particle size distribution is the most active catalyst (TOF of 136700 h^−1^). Alshammari et al. [15] found that the major factor determining catalyst activity is the particle size distribution. Au–Pd/MnO₂ with a narrower particle size distribution showed better activity than Au–Pd/MgO. The TOF values were 1874 and 1612 h^−1^ at 120 °C under 1 bar O_2_ for 1 h over Au–Pd/MnO₂ and Au–Pd/MgO, respectively. The catalytic activity of the gold-silver bimetallic-supported catalysts on ZIF-8 is lower than that of Au–Pd bimetallic-supported catalysts [15,47]. Cui et al. [48] found that the benzyl alcohol conversion and benzaldehyde selectivity were 17% and 97% over the Au@ZrO_2_ catalyst (the average gold particle sizes is 4.3 nm) at 40 °C within 7 h, respectively. The TOF value was 19 h^−1^ based on the amounts of converted benzyl alcohol and the total amount of gold. Compared with Au@ZrO_2_, although the average particle sizes of Au@Ag/ZIF-8, Ag@Au/ZIF-8, and AuAg/ZIF-8 were larger than that of the Au@ZrO_2_ catalyst, the catalysts give a higher TOF for the selective oxidation of benzyl alcohol. The benzaldehyde selectivities were 53.0%, 53.3%, and 35.2% within 1 h over Au@Ag/ZIF-8, Ag@Au/ZIF-8, and AuAg/ZIF-8, respectively. Core-shell Au@Ag and Ag@Au particles supported on ZIF-8 give higher benzaldehyde selectivity than cluster-structured catalyst AuAg/ZIF-8 in the selective oxidation of benzyl alcohol into benzaldehyde.

The effect of the solvent, reaction temperature, and reaction pressure on benzyl alcohol conversion and benzaldehyde selectivity in benzyl alcohol selective oxidation over Au@Ag/ZIF-8, Ag@Au/ZIF-8, and AuAg/ZIF-8 are shown in Table 1. Keeping the reactant and catalyst amounts unchanged, the conversion of benzyl alcohol and the selectivity of benzaldehyde were studied in various solvents at 130 °C under 8 bar O_2_ for 1 h. The solvent showed a strong influence on both benzyl alcohol conversion and benzaldehyde selectivity over Au@Ag/ZIF-8, Ag@Au/ZIF-8, and AuAg/ZIF-8 catalysts (Table 1, entries 1–5, 10–14, 19–23). For example, the conversions of benzyl alcohol in tetrahydrofuran, 1,4-dioxane, N,N-dimethylformamide (DMF), acetonitrile, and toluene were 65.7%, 23.7%, 69.1%, 27.7%, and 8.3% at 130 °C within 1 h over Au@Ag/ZIF-8, respectively (Table 1, entries 1–5). The benzaldehyde selectivities were 53.0%, 72.9%, 2.6%, 2.0%, and 29.3% in tetrahydrofuran, 1,4-dioxane, DMF, acetonitrile, and toluene over Au@Ag/ZIF-8, respectively (Table 1, entries 1–5). The highest catalytic activity (yield of 34.8%) was achieved in THF for the catalyst of Au@Ag/ZIF-8 in the selective oxidation of benzyl alcohol to benzaldehyde. Ag@Au/ZIF-8 and AuAg/ZIF-8 also showed the highest activity by using THF as the solvent for the selective oxidation of benzyl alcohol to benzaldehyde (Table 1, entries 10–14, 19–23). The yields of benzaldehyde were 40.1% and 26.0% over Ag@Au/ZIF-8 and AuAg/ZIF-8 in THF, respectively (Table 1, entries 10, 19). The benzyl alcohol conversion and benzaldehyde selectivity were found to be strongly dependent upon the reaction pressure and reaction temperature in the oxidation of benzyl alcohol (Table 1, entries 6–9, 15–18, 24–27). The conversion of benzyl alcohol increased with the increasing reaction pressure and reaction temperature in the oxidation of benzyl alcohol. However, an elevated reaction pressure and reaction temperature led to the decrease in benzaldehyde selectivity for all of the catalysts. The highest benzaldehyde yield was obtained at 130 °C under 8 bar O_2_ for all the catalysts. A good conversion of benzyl alcohol and good selectivity of benzaldehyde were achieved when the reaction was carried out at 130 °C in THF under 8 bar O_2_ within 1 h for all of the catalysts.

**Figure 8:**
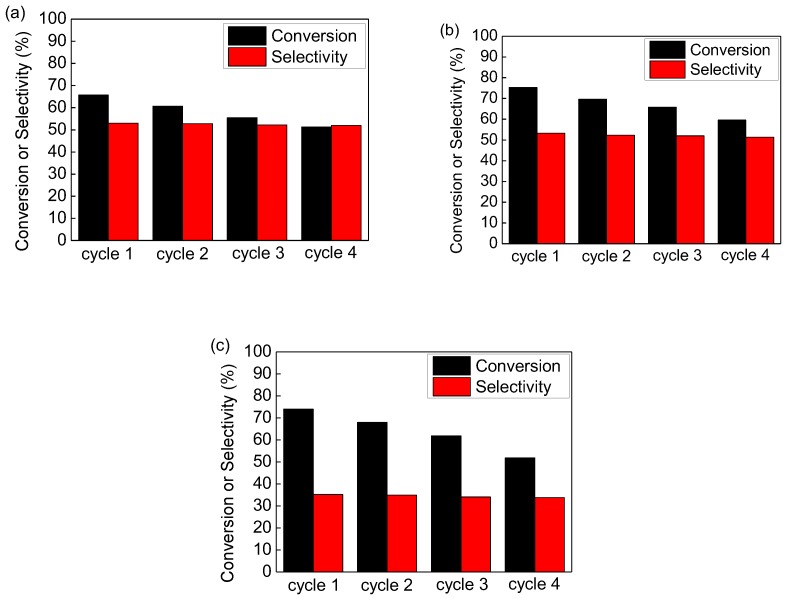
Recyclability of catalysts in the oxidation of benzyl alcohol: (**a**) Au@Ag/ZIF-8, (**b**) Ag@Au/ZIF-8, and (**c**) AuAg/ZIF-8 (reaction condition: benzyl alcohol (0.2 mmoL), THF (1.5 mL), and catalyst (0.04 g), 8 bar, 130 ^°^C, 1 h).

The reusability studies of Au@Ag/ZIF-8, Ag@Au/ZIF-8, and AuAg/ZIF-8 were carried out on the selective oxidation of benzyl alcohol in THF at 130 °C under 8 bar O_2_. The catalysts after reaction were recovered by centrifugation, washed with ethanol, and then dried at 40 °C for 3 h and reused for another reaction under the same conditions. The benzyl alcohol conversions and benzaldehyde selectivities in four consecutive cycles on the catalyst of Au@Ag/ZIF-8, Ag@Au/ZIF-8, and AuAg/ZIF-8 are shown in Figure 8. It has a slight decrease of the benzyl alcohol conversion in the second, third, and fourth reaction cycles for all of the catalysts, and no loss of benzaldehyde selectivity was detected during four consecutive cycles for the selective oxidation of benzyl alcohol. This result indicated that the catalysts have good stability, which is vital for traits for industrial prospects.

We performed a leaching test to examine whether there were any homogeneous active species in the solution that could catalyze the oxidation of benzyl alcohol. The benzyl alcohol oxidation was stopped after 0.5 h at 130 °C under 8 bar O_2_ using THF as the solvent. The conversions of benzyl alcohol reached 47.2%, 48.6%, and 42.6% over the Au@Ag/ZIF-8, Ag@Au/ZIF-8, and AuAg/ZIF-8 catalysts, respectively. After being cooled down to room temperature, the reaction solution was removed from the catalyst and transferred to another reactor under the same reaction conditions. The benzyl alcohol conversion increased by 2.2%, 2.6%, and 3.9% another 0.5 h after reaction, respectively. These results showed that there are some leaching active species under the reaction conditions.

## 4. Conclusions

In conclusion, we have successfully prepared core-shell and cluster structured Au@Ag, Ag@Au, and AuAg particles using the successive reduction method and physical mixing method, respectively. These particles were loaded on a zeolitic imidazolate framework-8 by the deposition-redispersion method to receive Au@Ag/ZIF-8, Ag@Au/ZIF-8, and AuAg/ZIF-8 catalysts. The most active catalyst was Ag@Au/ZIF-8 with a core-shell structure for the selective oxidation of benzyl alcohol to benzaldehyde, followed by Au@Ag/ZIF-8 and AuAg/ZIF-8. The core-shell structured catalysts Au@Ag/ZIF-8 and Ag@Au/ZIF-8 exhibited higher benzaldehyde selectivity than AuAg/ZIF-8 in the selective benzyl alcohol oxidation. The good catalytic activity was achieved when the reaction was carried out at 130 °C in THF under 8 bar O_2_ within 1 h for all of the catalysts. In addition, the catalysts can be readily recovered by centrifugation and reused for four consecutive cycles, thus making this procedure more environmentally acceptable.

## Figures and Tables

**Table 1:** The catalytic activity of Au@Ag/ZIF-8, Ag@Au/ZIF-8, and AuAg/ZIF-8 in the selective oxidation of benzyl alcohol to benzaldehyde ^a^.

Entry	Catalyst	Solvent	*T* (°C)	Pressure (bar)	Conv. (%) ^b^	S (%) ^c^	Yield (%) ^d^
1	Au@Ag/ZIF-8	THF	130	8	65.7	53.0	34.8
2	Au@Ag/ZIF-8	1,4-dioxane	130	8	23.7	72.9	17.3
3	Au@Ag/ZIF-8	DMF	130	8	69.1	2.6	1.8
4	Au@Ag/ZIF-8	Acetonitrile	130	8	27.7	2.0	0.6
5	Au@Ag/ZIF-8	Toluene	130	8	8.3	29.3	2.4
6	Au@Ag/ZIF-8	THF	130	10	77.0	24.9	19.2
7	Au@Ag/ZIF-8	THF	130	6	25.5	98.0	25.0
8	Au@Ag/ZIF-8	THF	120	8	40.1	66.7	26.7
9	Au@Ag/ZIF-8	THF	140	8	80.7	29.9	24.1
10	Ag@Au/ZIF-8	THF	130	8	75.3	53.3	40.1
11	Ag@Au/ZIF-8	1,4-dioxane	130	8	52.6	29.5	15.5
12	Ag@Au/ZIF-8	DMF	130	8	12.3	16.4	2.0
13	Ag@Au/ZIF-8	Acetonitrile	130	8	31.5	1.0	0.3
14	Ag@Au/ZIF-8	Toluene	130	8	1.0	96.5	1.0
15	Ag@Au/ZIF-8	THF	130	10	80.5	44.2	35.6
16	Ag@Au/ZIF-8	THF	130	6	34.0	96.5	32.8
17	Ag@Au/ZIF-8	THF	120	8	69.7	55.6	38.8
18	Ag@Au/ZIF-8	THF	140	8	80.0	40.2	32.2
19	AuAg/ZIF-8	THF	130	8	74.0	35.2	26.0
20	AuAg/ZIF-8	1,4-dioxane	130	8	51.9	35.0	18.2
21	AuAg/ZIF-8	DMF	130	8	4.9	81.9	4.0
22	AuAg/ZIF-8	Acetonitrile	130	8	2.0	95.0	1.9
23	AuAg/ZIF-8	Toluene	130	8	4.3	94.6	4.1
24	AuAg/ZIF-8	THF	130	10	80.6	30.6	24.7
25	AuAg/ZIF-8	THF	130	6	32.4	55.6	18.0
26	AuAg/ZIF-8	THF	120	8	34.7	62.3	21.6
27	AuAg/ZIF-8	THF	140	8	89.9	24.8	22.3

^a^ Reaction conditions: benzyl alcohol (0.2 mmoL), solvent (1.5 mL), and catalyst (0.04 g), 1 h; ^b^ Conversion was calculated by $C_{benzylalcohol}=\frac{n_{initial}-n_{afterreaction}}{n_{initial}}\times 100\%$; ^c^ Selectivity was calculated by $S=\frac{n_{benzyldehyde}}{n_{benzylalcohol-initial}\times C_{benzylalcohol}}\times 100\%$; ^d^ Yield was calculated by $Y=C_{benzylalcohol}\times S\times 100\%$.

## References

[1] Chen G.J., Wang J.S., Jin F.Z., Liu M.Y., Zhao C.W., Li Y.A., Dong Y.B. (2016). Pd@Cu(II)-MOF-catalyzed aerobic oxidation of benzylic alcohols in air with high conversion and selectivity. Inorg. Chem..

[2] Dai Y., Wu X.P., Tang Y., Yang Y., Gong X.Q., Fan J. (2016). Selectivity switching resulting in the formation of benzene by surface carbonates on ceria in catalytic gas-phase oxidation of benzyl alcohol. Chem. Commun..

[3] Narayanan S., Vijaya J.J., Sivasanker S., Alam M., Tamizhdurai P., Kennedy J. (2015). Characterization and catalytic reactivity of mordenite–investigation of selective oxidation of benzyl alcohol. Polyhedron.

[4] Nemanashi-Maumela M., Nongwe I., Motene R.C., Davids B.L., Meijboom R. (2017). Au and Ag nanoparticles encapsulated within silica nanospheres using dendrimers as dual templating agent and their catalytic activity. Mol. Catal..

[5] Kimi M., Jaidie M.M.H., Pang S.C. (2018). Bimetallic Cu-Ni nanoparticles supported on activated carbon for catalytic oxidation of benzyl alcohol. J. Phys. Chem. Solids.

[6] Kumar A., Gautam R.K., Belwal M. (2018). Synthesis and characterization of Au/γ-Al_2_O_3_ nanocatalysts for vapor-phase selective oxidation of benzyl alcohol under aerobic condition. Curr. Catal..

[7] Chen L.J., Yan J.Q., Tong Z.X., Yu S.Y., Tang J.T., Ou B.L., Yue L.J., Tian L. (2018). Nanofiber-like mesoporous alumina supported palladium nanoparticles as a highly active catalyst for base-free oxidation of benzyl alcohol. Microporous Mesoporous Mater..

[8] Sun J.Y., Tong X.L., Liu Z.H., Liao S.Y., Zhang X.L., Xue S. (2016). Gold-catalyzed selectivity-switchable oxidation of benzyl alcohol in the presence of molecular oxygen. Catal. Commun..

[9] Song Y.J., Jesús Y.M.L.D., Fanson P.T., Williams C.T. (2013). Preparation and characterization of dendrimer-derived bimetallic Ir–Au/Al_2_O_3_ catalysts for CO oxidation. J. Phys. Chem. C.

[10] Davis S.E., Ide M.S., Davis R.J. (2013). Selective oxidation of alcohols and aldehydes over supported metal nanoparticles. Green Chem..

[11] Bhattacharya C., Jagirdar B.R. (2018). Monodisperse colloidal metal nanoparticles to core-shell structures and alloy nanosystems via digestive ripening in conjunction with solvated metal atom dispersion: A mechanistic study. J. Phys. Chem. C.

[12] Rostek A., Breisch M., Loza K., Garcia P.R.A.F., Oliveira C.L.P., Prymak O., Heggen M., Köller M., Sengstock C., Epple M. (2018). Wet-chemical synthesis of Pd-Au core-shell nanoparticles (8 nm): From nanostructure to biological properties. Chem. Sel..

[13] Sun J.Y., Han Y.X., Fu H.Y., Qu X.L., Xu Z.Y., Zheng S.R. (2017). Au@Pd/TiO_2_ with atomically dispersed Pd as highly active catalyst for solvent-free aerobic oxidation of benzyl alcohol. Chem. Eng. J..

[14] Henning A.M., Watt J., Miedziak P.J., Cheong S., Santonastaso M., Song M., Takeda Y., Kirkland A.I., Taylor S.H., Tilley R.D. (2013). Gold-palladium core-shell nanocrystals with size and shape control optimized for catalytic performance. Angew. Chem. Int. Ed..

[15] Alshammari H., Alhumaimess M., Alotaibi M.H., Alshammari A.S. (2017). Catalytic activity of bimetallic AuPd alloys supported MgO and MnO_2_ nanostructures and their role in selective aerobic oxidation of alcohols. J. King Saud Univ. Sci..

[16] Wang Z.J., Balkus K.J. (2018). Wrinkled mesoporous carbon supported Pd nanoparticles for hydrogenation and aerobic oxidation reactions. J. Porous Mater..

[17] Kumar A., Screedhar B., Chary K.V.R. (2015). Highly dispersed gold nanoparticles supported on SBA-15 for vapor phase aerobic oxidation of benzyl alcohol. J. Nanosci. Nanotechnol..

[18] Dhakshinamoorthy A., Asiri A.M., Garcia H. (2017). Metal organic frameworks as versatile hosts of Au nanoparticles in heterogeneous catalysis. ACS Catal..

[19] Huang Y.B., Liang J., Wang X.S., Cao R. (2017). Multifunctional metal-organic framework catalysts: Synergistic catalysis and tandem reactions. Chem. Soc. Rev..

[20] Zhu Y., Wang Y.M., Liu P., Wu Y.L., Wei W., Xia C.K., Xie J.M. (2015). Cage-like pores of a metal-organic framework for separations and encapsulation of Pd nanoparticles for efficient catalysis. New J. Chem..

[21] To T.A., Tran C.B., Nguyen N.T.H., Nguyen H.H.T., Nguyen A.T., Phan A.Q., Phan N.T.S. (2018). An efficient access to b-ketosulfones via bsulfonylvinylamines: Metal-organic framework catalysis for the direct C–S coupling of sodium sulfinates with oxime acetates. RSC Adv..

[22] Thornburg N.E., Liu Y., Li P., Hupp J.T., Farha O.K., Notestein J.M. (2016). MOFs and their grafted analogues: Regioselective epoxide ring-opening with Zr_6_ nodes. Catal. Sci. Technol..

[23] Noori Y., Akhbari K. (2017). Post-synthetic ion-exchange process in nanoporous metal-organic frameworks: An effective way for modulating their structures and properties. RSC Adv..

[24] Canivet J., Aguado S., Schuurman Y., Farrusseng D. (2013). MOF-supported selective ethylene dimerization single-site catalysts through one-pot postsynthetic modification. J. Am. Chem. Soc..

[25] Zhang Y.G., Ying J.Y. (2015). Main-chain organic frameworks with advanced catalytic functionalities. ACS Catal..

[26] Chughtai A.H., Ahmad N., Younus H.A., Laypkov A., Verpoort F. (2015). Metal-organic frameworks: Versatile heterogeneous catalysts for efficient catalytic organic transformations. Chem. Soc. Rev..

[27] Guo Z., Xiao C., Maligal-Ganesh R.V., Zhou L., Goh T.W., Li X., Tesfagaber D., Thiel A., Huang W. (2014). Pt nanoclusters confined within metal-organic framework cavities for chemoselective cinnamaldehyde hydrogenation. ACS Catal..

[28] Li X., Guo Z., Xiao C., Goh T.W., Tesfagaber D., Huang W. (2014). Tandem catalysis by palladium nanoclusters encapsulated in metal-organic frameworks. ACS Catal..

[29] Neelakanda P., Barankova E., Peinemann K.V. (2016). Polymer supported ZIF-8 membranes by conversion of sputtered zinc oxide layers. Microporous Mesoporous Mater..

[30] Wang Y.W., Zeng Y.Y., Wu X.C., Mu M.M., Chen L.G. (2018). A novel Pd-Ni bimetallic synergistic catalyst on ZIF-8 for Sonogashira coupling reaction. Mater. Lett..

[31] Xia B.Q., Cao N., Dai H.M., Su J., Wu X.J., Luo W., Cheng G.Z. (2014). Bimetallic nickel–rhodium nanoparticles supported on ZIF-8 as highly efficient catalysts for hydrogen generation from hydrazine in alkaline solution. ChemCatChem.

[32] Amarante S.F., Freire M.A., Mendes D.T.S.L., Freitas L.S., Ramos A.L.D. (2017). Evaluation of basic sites of ZIFs metal organic frameworks in the Knoevenagel condensation reaction. Appl. Catal. A Gen..

[33] Hwang Y.K., Hong D.Y., Chang J.S., Jhung S.H., Seo Y.K., Kim J., Vimont A., Daturi M., Serre C., Férey G. (2008). Amine grafting on coordinatively unsaturated metal centers of MOFs: Consequences for catalysis and metal encapsulation. Angew. Chem. Int. Ed..

[34] Zhou K., Mousavi B., Luo Z., Phatanasri S., Chaemchuen S., Verpoort F. (2017). Characterization and properties of Zn/Co zeolitic imidazolate frameworks vs. ZIF-8 and ZIF-67. J. Mater. Chem. A.

[35] Villa A., Wang D., Su D.S., Prati L. (2009). Gold sols as catalysts for glycerol oxidation: The role of stabilizer. Chem. Catal. Chem..

[36] Ding C., Zhang X.R., Li C.C., Hao X.G., Wang Y.H., Guan G.Q. (2016). ZIF-8 incorporated polyether block amide membrane for phenol permselective pervaporation with high efficiency. Sep. Purif. Technol..

[37] Ismail K., Gérald C., Habiba N., Guillaume O., Claire M., Joël P. (2016). Assessment of the energetic performances of various ZIFs with SOD or RHO topology using high pressure water intrusion-extrusion experiments. Dalton Trans..

[38] Misra M., Singh N., Gupta R.K. (2017). Enhanced visible-light-driven photocatalytic activity of Au@Ag core-shell bimetallic nanoparticles immobilized on electrospun TiO_2_ nanofibers for degradation of organic compounds. Catal. Sci. Technol..

[39] Pramanik S., Chattopadhyay S., Das J.K., Manju U., De G. (2016). Extremely fast Au–Ag alloy-dealloy associated reversible plasmonic modifications in SiO_2_ films. J. Mater. Chem. C.

[40] Butova V.V., Budnyk A.P., Bulanova E.A., Lamberti C., Soldatov A.V. (2017). Hydrothermal synthesis of high surface area ZIF-8 with minimal use of TEA. Solid State Sci..

[41] Singh A.K., Xu Q. (2013). Metal-organic framework supported bimetallic Ni-Pt nanoparticles as high-performance catalysts for hydrogen generation from hydrazine in aqueous solution. ChemCatChem.

[42] Liu L.L., Tai X.S., Zhang N.N., Meng Q.G., Xin C.L. (2016). Supported Au/MIL-53(Al): A reusable green solid catalyst for the three-component coupling reaction of aldehyde, alkyne, and amine. React. Kinet. Mech. Cat..

[43] Liu L.L., Tai X.S., Liu J.B., Li D., Zhou X.J., Zhang L.J., Wei X.F. (2018). Preparation of propargylamines catalyzed by heterogeneous catalysts with double catalytic sites. Chem. J. Chin. Univ..

[44] Yong G.P., Tian D., Tong H.W., Liu S.M. (2010). Mesoporous SBA-15 supported silver nanoparticles as environmentally friendly catalysts for three-component reaction of aldehydes, alkynes and amines with glycol as a “green” solvent. J. Mol. Catal. A Chem..

[45] Galvanin F., Sankar M., Cattaneo S., Bethell D., Dua V., Hutchings G., Gavriilidis A. (2018). On the development of kinetic models for solvent-free benzyl alcohol oxidation over a gold-palladium catalyst. Chem. Eng. J..

[46] Philip A., Lihavainen J., Keinänen M., Pakkanen T.T. (2017). Gold nanoparticle-decorated halloysite nanotubes—Selective catalysts for benzyl alcohol oxidation. Appl. Clay Sci..

[47] Miedziak P., Sankar M., Dimitratos N., Lopez-Sanchez J.A., Carley A.F., Knight D.W., Taylor S.H., Kiely C.J., Hutchings G.J. (2011). Oxidation of benzyl alcohol using supported gold–palladium nanoparticles. Catal. Today.

[48] Cui W.J., Xiao Q., Sarina S., Ao W.L., Xie M.X., Zhu H.Y., Bao Z. (2014). Au–Pd alloy nanoparticle catalyzed selective oxidation of benzylalcohol and tandem synthesis of imines at ambient conditions. Catal. Today.

